# Supplementary material for: Comparative genomics provides new insights into the diversity, physiology, and sexuality of the only industrially exploited tremellomycete: Phaffia rhodozyma
Source: BMC Genomics. 2016 Nov 9;17:901. doi: 10.1186/s12864-016-3244-7 (PMC5103461; doi:10.1186/s12864-016-3244-7)
Supplement: Additional file 6: — List of orphan genes with links to PFAM (related to Additional file 1: Table S1). (ZIP 1428 kb) [file 12864_2016_3244_MOESM6_ESM.zip › PFAM_UNIQUE/G01776_P.html]

#                                                                            --- full sequence --- -------------- this domain -------------   hmm coord   ali coord   env coord
  
# target name        accession   tlen query name           accession   qlen   E-value  score  bias   #  of  c-Evalue  i-Evalue  score  bias  from    to  from    to  from    to  acc description of target
  
#------------------- ---------- ----- -------------------- ---------- ----- --------- ------ ----- --- --- --------- --------- ------ ----- ----- ----- ----- ----- ----- ----- ---- ---------------------
  
G01776\_P             -            277 Baculo\_PEP\_C         PF04513.7    140      0.04   12.4  23.3   1   3      0.87   2.3e+02    0.2   2.1    71    92    54    75    48   115 0.58 - Baculovirus polyhedron envelope protein, PEP, C terminus  
G01776\_P             -            277 Baculo\_PEP\_C         PF04513.7    140      0.04   12.4  23.3   2   3   0.00039       0.1   11.1  15.8    30   137   109   221    68   224 0.84 - Baculovirus polyhedron envelope protein, PEP, C terminus  
G01776\_P             -            277 Baculo\_PEP\_C         PF04513.7    140      0.04   12.4  23.3   3   3     0.055        14    4.1   0.1    37    80   223   266   214   275 0.69 - Baculovirus polyhedron envelope protein, PEP, C terminus  
G01776\_P             -            277 BcsB                 PF03170.8    605     0.029   11.0   0.4   1   1   7.8e-06     0.047   10.4   0.4   482   581     9   105     7   123 0.71 - Bacterial cellulose synthase subunit  
G01776\_P             -            277 HAP                  PF03866.8    164       9.1    4.7  14.3   1   3     0.019   1.1e+02    1.2   0.5    96   137    41    83    14   109 0.63 - Hydrophobic abundant protein (HAP)  
G01776\_P             -            277 HAP                  PF03866.8    164       9.1    4.7  14.3   2   3    0.0092        55    2.2   6.5    26   117    96   194    80   213 0.70 - Hydrophobic abundant protein (HAP)  
G01776\_P             -            277 HAP                  PF03866.8    164       9.1    4.7  14.3   3   3   0.00084         5    5.6   0.2    38    69   232   263   205   272 0.81 - Hydrophobic abundant protein (HAP)  
G01776\_P             -            277 Ins\_allergen\_rp      PF06757.8    179     0.028   12.6   0.0   1   1   8.2e-06     0.049   11.8   0.0    51   122    44   127    39   152 0.73 - Insect allergen related repeat, nitrile-specifier detoxification  
G01776\_P             -            277 Staph\_haemo          PF05480.6     43       9.9    4.6   9.6   1   4      0.13   7.5e+02   -1.4   0.1    24    33    79    88    55    90 0.52 - Staphylococcus haemolytic protein  
G01776\_P             -            277 Staph\_haemo          PF05480.6     43       9.9    4.6   9.6   2   4     0.011        64    2.0   0.1     5    19   124   141   121   155 0.71 - Staphylococcus haemolytic protein  
G01776\_P             -            277 Staph\_haemo          PF05480.6     43       9.9    4.6   9.6   3   4       0.4   2.4e+03   -3.0   0.1    34    38   214   218   205   222 0.56 - Staphylococcus haemolytic protein  
G01776\_P             -            277 Staph\_haemo          PF05480.6     43       9.9    4.6   9.6   4   4   0.00031       1.9    7.0   0.3    26    41   232   247   230   249 0.90 - Staphylococcus haemolytic protein  
G01776\_P             -            277 TMV\_coat             PF00721.16   152      0.63    8.6   5.7   1   2     0.022        43    2.7   0.6    78   126    61   108    50   145 0.64 - Virus coat protein (TMV like)  
G01776\_P             -            277 TMV\_coat             PF00721.16   152      0.63    8.6   5.7   2   2   0.00085       1.7    7.2   0.5    26    54   183   211   181   243 0.84 - Virus coat protein (TMV like)
